# Hope and life satisfaction among Chinese shadow education tutors: The mediating roles of positive coping and perceived social support

**DOI:** 10.3389/fpsyg.2022.929045

**Published:** 2022-08-23

**Authors:** Jie Ji, Linzhi Zhou, Yunpeng Wu, Mohan Zhang

**Affiliations:** ^1^Jing Hengyi School of Education, Hangzhou Normal University, Hangzhou, China; ^2^School of Teacher Education, Dezhou University, Dezhou, China

**Keywords:** hope, positive coping, perceived social support, life satisfaction, mediation

## Abstract

Previous studies of the relationship between hope and life satisfaction left the underlying mechanism of how hope predicts life satisfaction unexplored to scholars. This study thus investigates the two potential mediators in the relationship between hope and life satisfaction among a sample of Chinese shadow education institution (SEI) tutors who may be under immense professional development pressure from a cross-sectional approach. The main body of the study consists of an online survey in which 221 SEI tutors reported their hope, positive coping, perceived social support, and life satisfaction. The survey results were analyzed using mediation and moderation analysis *via* SPSS 23.0. The results indicated that positive coping improved the relationship between hope and life satisfaction, supporting the hypothesis regarding the serial mediating effect of positive coping and perceived social support. In other words, tutors with a high level of hope tend to adopt positive coping strategies, thus will receive more social support and improve life satisfaction. Our findings revealed the independent and accumulative mediating effects of positive coping and perceived social support on the relationship between hope and life satisfaction, and had implications for the psychological intervention of SEI tutors who are currently facing enormous industry pressure.

## Introduction

Shadow education which provides private tutoring for profit outside of standard school hours, has become a universal and inevitable trend in modern times. East Asian countries are the major markets for shadow education as they were known as the “cradle of private tutoring.” With the emergence of large collectivized shadow education institutions (SEIs) in the market, Chinese shadow education has developed and expanded rapidly since the early age of the 21st century. According to the *Chinese 4-Year College Graduates’ Employment Annual Report* issued in 2020, compulsory education and SEI tutoring sectors witnessed growth in employees as the proportion of graduates employed in private compulsory education and SEIs is 7.6%, surpassing the proportion of graduates employed in public education, which is 6.1% (MyCOS Institute, 2020). Furthermore, China has the world’s largest school system and most extensive shadow provision (Zhang and Bray, 2021). Shadow education has caught much attention for its influences on the core domains of schools and schooling, such as the curriculum, academic performance, education policy, and even the maturing global education revolution (Baker, 2020; Hajar and Karakus, 2022). Studies about shadow education in the domain of educational psychology paid attention to parents’ anxiety and students’ mental health (Park et al., 2011; Carr and Wang, 2018), yet there is a massive gap in the tutors’ mental status and attitude toward shadow education. For instance, Bray (2021) noted that the list of possible themes for the investigation of shadow education could be as long as that of regular education, and shadow education deserves much more attention from analysts of teachers to better document and understand shadow education.

Regarding mental status, an impressive volume of research focused on public school teachers. Bano and Malik (2014) indicated that teachers’ life satisfaction would be low when facing tremendous workplace stress in public or private educational institutions. Unlike teachers in public schools, the SEI tutors as a part of private education were provided by commercial firms, which has the property of market orientation. As shadow education increases family educational costs, students’ academic burden, and education inequality (Park et al., 2011; Zhang and Bray, 2018), the Chinese government has introduced multiple regulatory measures for SEIs. The measures included limiting the scale, time, and cost of the academic subjects tutoring in SEIs in the recent 10 years to relieve students’ burden and promote education equalization. Under the impact of COVID-19, the SEI tutoring industry has shrunk rapidly in China for gatherings other than regular schooling were restricted, and most face-to-face tutoring sessions were suspended, causing turmoil to SEI and unemployment risks. Tutors confront to tremendous challenges in income and career development, the life satisfaction of Chinese SEI tutors is under great shock, and countless tutors had to choose an alternative profession.

Based on the Conservation of Resources Theory (Hobfoll et al., 2018), uncertainty leads to a lack of resources invested in regulating emotions and maintaining stability, which results in psychological distress and emotional exhaustion (Godinić et al., 2020). Given that the decreased life satisfaction may cause negative attitudes and behaviors of SEI tutors toward the shadow education industry, the mental health of SEI tutors is an essential criterion under the background of vigorously promoting educational reform to promote the sustainable development of shadow education. Having SEI tutors’ mental health under the double shocks of COVID-19 and regulation in mind, this study built a theoretical model in which hope and life satisfaction are mediated by positive coping and perceived social support based on the empirical study of Chinese SEI tutors in the post-COVID-19 era. We try to verify whether SEI tutors still hold a positive attitude and believe that hard work can lead to success and still have high life satisfaction in hard times. By focusing on the psychological issues of Chinese SEI tutors, this study will contribute to shadow education literature in the geographic coverage and in the specific themes.

## Literature review

### The relationship between hope and life satisfaction

As a character and cognitive set, hope is not only a critical factor in positive psychology but also an antecedent of positive affect (Ciarrochi et al., 2015). Hope theory believes that the one with hope knows the pathways to achieve goals and has the agency to make goals come into fruition (Snyder et al., 2002). Hope was interpreted as actuating thinking and alternative ways of thinking, and correlated with personal adaptation (Karataş et al., 2021). Therefore, hope is especially valued in adversity. When individuals are under severe stress, hope plays a more important role in coping with the stress (Lopez et al., 2000). Research has shown that hope was associated with positive psychological and health-related outcomes in adults and children coping with various adverse events (Barnum et al., 1998). However, more research is needed to explore its potential mechanism in the future.

Life satisfaction, covering both cognitive and emotional assessments, refers to the degree to which a person positively evaluates the overall quality of life, and acts as the dominant indicator of happiness (Diener et al., 1985). Previous research has shown that wealth, health, job satisfaction, and entertainment are closely correlated with life satisfaction, and thus life satisfaction of education workers can be valued on income level, professional status, environmental conditions, and personality and characteristics (Kanadli et al., 2022). Although there is an absence of direct report of SEI tutors’ level of life satisfaction, the Chinese Society of Education’s Survey (Chinese Society of Education, 2016) illustrated that SEI tutors face financial and mental pressures. Tutoring is described as a semi-profession in terms of professional development (Bray, 2021), which lacks the occupational stability. The profit-making nature of SEI makes it impossible to provide tutors with professional development and training systems, and they seem to reach the glass ceiling for their career prospects. Financially, tutors do not have a solid salary system as public school teachers, their social security system is far more than sound, and the industry shrank puts SEI tutors at financial risk. Therefore, it is reasonable to assume that tutors’ job and life satisfaction declined in the post-COVID-19 era when they experienced income and identity crises.

Hope and life satisfaction are two cognitive well-being factors (Jiang et al., 2020). The effect between hope and life satisfaction is reciprocal. On one hand, hope emerged as the highest correlating character strength with life satisfaction after investigating the level of 24 character strengths in eight occupational groups and six age groups (Heintz and Ruch, 2019). On the other hand, teachers who have greater life satisfaction and experience more positive emotions tend to possess higher levels of strength of hope (Chan, 2009). The cross-sectional study has shown that hope strongly influences life satisfaction, and a positive relationship between hope and life satisfaction exists in both adults and adolescents, especially the subgroups who face difficulties (Proctor et al., 2011). Longitudinal studies have further demonstrated that after controlling for initial life satisfaction, hope is a significant predictor of later life satisfaction (Marques et al., 2013). Hope mediates the relationship of life satisfaction with various situational and personality traits, and plays a moderating effect between adults’ established goals and life satisfaction (Satici, 2016; Nie et al., 2019). Recent studies in the context of COVID-19 highlight that hope contributes positively to individuals’ life satisfaction (Karataş et al., 2021). However, Chen et al. (2021) proposed that hope was not significantly associated with life satisfaction based on the empirical study of Chinese ethnic minority college students. The inconsistent findings remind us that some uncertain background variables may regulate the relationship between hope and life satisfaction. While studies about hope and life satisfaction focus on various working populations, few have explored the mechanism between hope and life satisfaction among SEI tutors. Hence, this study will focus on this particular topic.

### Hope, positive coping, perceived social support, and life satisfaction

Although hope has been demonstrated to be positively associated with life satisfaction, little has been known about the mechanisms behind the link between hope and life satisfaction (Du et al., 2015). Studies based on adolescent samples found that school connectedness and perceived community support mediated the relationship between hope and life satisfaction, hopefully mediates the association between family support and meaningful instrumental activity (You et al., 2008; Ng et al., 2014; Jiang et al., 2020). Research on predicting life satisfaction highlights the importance of perceived social support and positive coping (Li et al., 2021). However, the potential mediator variables underlying the relationship between adults’ hope and life satisfaction need further exploration. Therefore, this study investigates the mechanism by which how the two psychosocial factors, perceived social support and positive coping, influence SEI tutors’ well-being.

Perceived social support refers to the perception that one is cared for by others and has a reliable social network. Family, friends, colleagues, and significant others are all the possible sources of social support (Taylor, 2011). Social support comprises structural support and functional support. Structural support focuses on the number of people who can help, psychological intimacy, physical accessibility, etc. Functional support includes the following dimensions: emotional support, informational support, tangible support, positive social interaction, and affective support (Azañedo et al., 2021). According to attachment theory and intimacy theory, the mental well-being benefits of perceived social support have been identified as interpersonal satisfaction and perceived social support positively affects life satisfaction. When necessary, individuals’ access to social support networks can meet their internal needs for belonging and acceptance (Nezlek, 2001). Wilson et al. (2020) found that perceived social support was significantly associated with greater mindfulness, self-compassion, and positive psychological outcomes (such as life satisfaction). Individuals with adequate social support are more likely to be satisfied with life in both collectivistic and individualistic cultures (Koydemir et al., 2013). Hope, as a positive emotional trait, stimulates prosocial behaviors that help build and maintain supportive social relationships, hope has a positive predictive effect on social support, and improves individuals’ perceived social support (Wang et al., 2018), people with hope tend to actively seek social support and increase the usage of social support.

Coping is broadly defined as a person’s cognition and efforts to deal with internal and external needs beyond personal resources (Folkman et al., 1986). Positive coping, also known as problem-focused coping, reflects an individual’s efforts to assemble resources that help realize the achievement of challenging goals and personal growth (Schwarzer and Knoll, 2003). Previous research has suggested that positive coping styles promote personal growth, thereby increasing life satisfaction (Li et al. 2016). Hope, viewed as either a trait or a state, was considered the factor in explaining the relationship between coping style and life satisfaction, and Folkman (2010) suggested that the relationship between hope and coping is dynamic and interdependent, each, in turn, supports and is supported by the other. Studies concluded that hope was seen as an effective coping resource against the negative feelings of depression and despair, and individuals who have a higher level of hope generate more coping strategies to cope with setbacks and thus experience more positive emotions that increase their life satisfaction (Reichard et al., 2013). According to the moderated and mediation model, Swanson et al. (2018) have pointed out that positive coping mediates the relationship between hope and perceived social support. Positive and problem-focused coping styles positively moderate perceived social support (Dunkley et al., 2016). Although many studies have demonstrated that positive coping strategies can predict and mediate different types of social support (Zamanian et al., 2021), no research has directly supported the mediating effect of positive coping-perceived social support between hope and life satisfaction. Thus, based on the above arguments, we assumed that positive coping is another potential mediator between hope and life satisfaction.

### Current study

**Figure 1:**
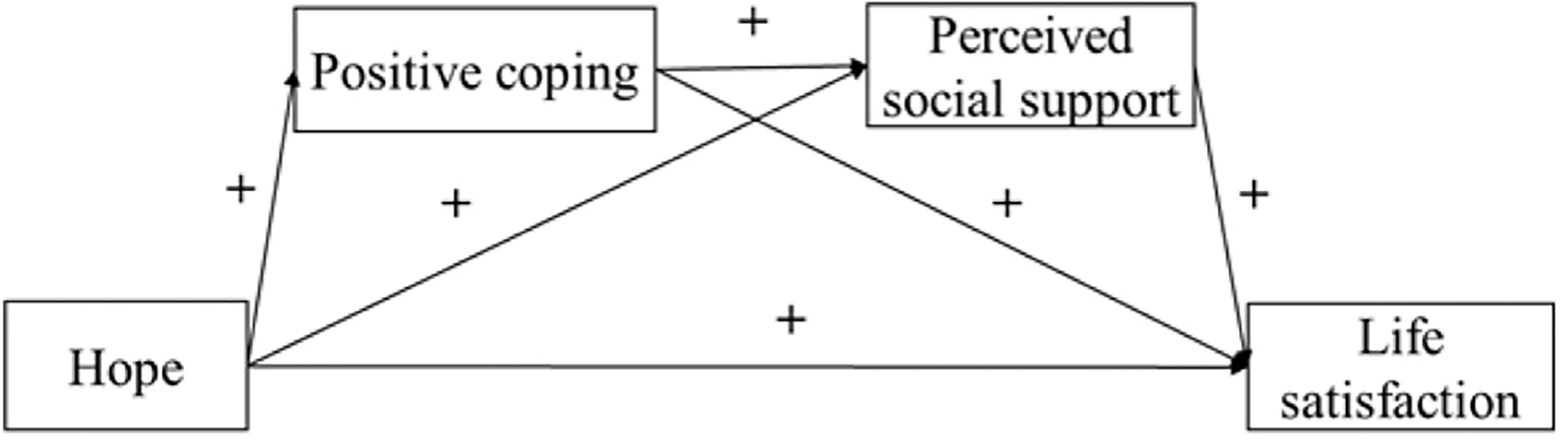
The proposed model.

Based on the existing literature, this study aims to investigate the effect of hope on life satisfaction and its underlying mechanisms based on the empirical study of Chinese SEI tutors. Toward this end, we propose a model (Figure 1) and seek to answer (a) whether positive coping and perceived social support mediates the association between hope and life satisfaction, respectively, and (b) whether hope is associated with life satisfaction through the chain-mediated mediating role of positive coping and perceived social support. Therefore, we propose the following hypotheses:

*Hypothesis* 1: Hope is positively associated with life satisfaction.

*Hypothesis* 2: Perceived social support mediates the relationship between hope and life satisfaction.

*Hypothesis* 3: Hope is positively associated with life satisfaction through the mediation of positive coping.

*Hypothesis* 4: Hope positively predicts life satisfaction through the chain intermediary of positive coping and perceived social support.

This study collects cross-sectional data from Chinese SEI tutors to verify the above hypotheses. The study aims to contribute to the design of interventions in life satisfaction of SEI tutors by adopting positive coping styles and promoting the sustainable development of the shadow education industry. In addition, the findings will provide empirical evidence for life satisfaction interventions for policymakers and SEIs and provide more social support for education workers, especially tutors themselves.

## Materials and methods

### Participants and procedures

This research got the approval of the Institutional Ethics Committee of Hangzhou Normal University. We advertised the survey through the internet, and simultaneously sent the link of the questionnaire to the SEI tutors through a convenience sampling method at the same time. Tutorial companies favor densely populated areas for higher enrolments (Zhang and Bray, 2021), and most of the participants in this study were recruited from Hangzhou, the capital city of Zhejiang province, which is one of the most important metropolises in China. The period of data collection lasted 14 days in November 2021. The SEI tutors were informed of the purpose of the study and their autonomy before the survey. Electronic informed consent forms were obtained from each participant, the participants were noted free to withdraw from the study at any time. Once recruited and consented, the participants completed the survey through the Wenjuanxing platform,^1^ a prominent online survey tool in China. The survey was conducted anonymously to ensure honest responses and full respect and protection of individual privacy rights before, during, and after the data collection process. It took an average of 15–20 min for a participant to complete this questionnaire. We collected a final sample of 223 participants. After eliminating missing values, the final dataset contains 221 responses, resulting in a 99.1% response rate.

Participants in this study involved 221 SEI tutors (Table 1). The age group of the participants was distributed as follows: 25 years and below (*n* = 38, 17%), 26–35 years (*n* = 130, 59%), 36–45 years (*n* = 38, 17%), and 46 years and above (*n* = 15, 7%). For education background, most of the participants (*n* = 164, 74%) reported holding a bachelor’s degree, 20 participants (9%) held a master’s or above degree, and 37 participants (17%) had completed three-year college and below education. For work experience, most of the participants (*n* = 120, 54%) worked in SEI for 1–5 years, 26 participants (12%) worked in SEI for no more than 1 year, 42 participants (19%) worked in SEI for 6–10 years, and 33 participants (15%) worked in SEI more than 10 years. As for the monthly salary, 68 participants’ income (31%) was no more than RMB 4,999 yuan, 99 (45%) were between RMB 5,000 and 9,000 yuan, 34 (15%) were between RMB 10,000 and 14,999 yuan, and 20 (9%) were more than RMB 15,000 yuan. The participants were asked to describe the scope of their institution’s business. In summary, 81 participants (37%) replied academic subject tutoring; 90 participants (41%) replied academic subject tutoring and non-academic tutoring, and the former being the dominant one; 27 participants (12%) replied academic subject tutoring and non-academic tutoring, with the latter being the dominant one; 23 participants (10%) replied non-academic tutoring.

**Table 1:** Demographic statistics (*N* = 221).

Variables	Frequency (*N*)	Percent (%)
*Gender*		
Female	152	69
Male	69	31
*Age group*		
25 years and below	38	17
26–35 years	130	59
36–45 years	38	17
46 years and above	15	7
*Education*		
Higher vocational colleges	37	17
University/Bachelor’s	164	74
Master’s and above	20	9
*Work experience*		
Less than 1 year	26	12
1–5 years	120	54
6–10 years	42	19
10 years and above	33	15
*Monthly salary*		
RMB 4,999 and below	68	31
RMB 5,000–9,999	99	45
RMB 10,000–14,999	34	15
RMB 15,000 and above	20	9

### Measures

#### Hope

The Chinese version of the Adult Dispositional Hope Scale (ADHS) is a 12-item self-report questionnaire used to assess the hope level, which includes agencies (4 items, e.g., “I meet the goals that I set for myself”) and pathway (4 items, e.g., “I can think of many ways to get out of a jam”). Each item is rated on a four-point Likert scale, ranging from 1 (completely disagree) to 4 (completely agree). The total score range from 8 to 32. A higher score indicates a higher degree of sense of hope. The Cronbach’s alpha is 0.78 in the present study.

#### Social support

Perceived social support was measured by the Perceived Social Support Scale (PSSS; Zimet et al., 1988). Validated in the Chinese context by Li et al. (2017), PSSS is a valid instrument for upholding concurrent and construct validity. To specify, PSSS is a 12-item self-report scale that assesses perceived support arising from three dimensions. The supports assessed are as follows: (1) Family support (4 items, e.g., “I get the emotional help and support I need from my family”), (2) Friend support (4 items, e.g., “I can count on my friends when things go wrong”), and (3) Others support (4 items, e.g., “There is a special person in my life who cares about my feelings”). Each item is scored on a seven-point scale ranging from 1 (completely disagree) to 7 (completely agree). Thus, total scores range from 12 to 84, which are applied as indicators of perceived social support, with higher scores indicating higher perceived social support. The Cronbach’s alpha is 0.97 in this study.

#### Positive coping

The Trait Coping Style Questionnaire (TCSQ) is used to evaluate the coping style of the subjects in response to stressful emotions in life (Wang and Wang, 1999). TCSQ consists of two subscales: negative coping (NC) and positive coping (PC), and each subscale has ten items. Each item is rated on a five-point Likert scale from 1 (definitely yes) to 5 (definitely not). The questionnaire has good reliability and validity support and could reflect the health-related coping style with individual trait attributes. This study employs the PC subscale. A higher score indicates that the participant is more likely to utilize positive coping strategies. The Cronbach’s alpha is 0.81 in this study.

#### Life satisfaction

The Satisfaction with Life Scale (SWLS) in the extant study was compiled by Diener et al. (1999). The Chinese version of the SWLS has been proved to be an effective tool for measuring the life satisfaction of the general population (Ye, 2017). The scale includes five items (life is close to ideal, living conditions are good, life is satisfied, getting what you want, affirming the life path), which is used to assess satisfaction with the respondent’s life as a whole (Diener et al., 1999). Each item is rated on a seven-point Likert scale from 1 (definitely yes) to 7 (definitely not), the total scores range from 5 to 35. Mean total scores are used to indicate the level of life satisfaction. Higher total scores indicate higher life satisfaction. The Cronbach’s alpha is 0.93 in the current study, which shows good reliability and validity.

### Statistical analysis

All data were analyzed using SPSS 23.0 (IBM, Chicago, IL, United States). Categorical data were reported as frequencies while continuous data, as mean values. We applied Hayes’ (2013) SPSS macro PROCESS (Model 6) to test the mediating effect. The bootstrapping method with robust standard errors was employed to test the significance of the effects (Hayes, 2013). The bootstrapping method produced 95% bias-corrected confidence intervals (CIs) of these effects from 5,000 resamples of the data. If CIs did not include zero, the effects in Model 6 were significant at *α* = 0.05. All statistical tests were two-tailed.

## Results

### Descriptive statistics and correlations

The four questionnaires were tested for common method biases by factor analysis. By exploratory factor analysis, eight factors with eigenvalues over 1 were obtained. The first factor accounted for 36.39% of the total variance. The data suggested that the common method biases were not significant.

As shown in Table 2, hope was significantly and positively associated with positive coping, social support, and life satisfaction. Positive coping was significantly and positively related to social support and life satisfaction. Social support was significantly and positively associated with life satisfaction. It is worth noting that the average life satisfaction is 19.83. According to Pavot and Diener (2009), a score of 20 represents the neutral point on the SWLS. Thus, SEI tutors appear dissatisfied with their lives, partially explaining the employees’ current life satisfaction.

**Table 2:** Means, standard deviations, and correlation coefficients for key variables.

	M	SD	1	2	3	4
1. Hope	21.66	4.34	1			
2. Positive coping	34.41	7.23	0.662^**^	1		
3. Social support	56.36	15.97	0.546^**^	0.702^**^	1	
4. Life satisfaction	19.83	6.66	0.650^**^	0.651^**^	0.613^**^	1

** *p* < 0.01.

### Testing for the mediation effects

The previous analyses revealed that hope, positive coping, and perceived social support were correlated with life satisfaction. First, we conducted the regression analyses before testing the mediation model to explore the paths between the study variables. All these correlated variables were included in the regression analyses. As shown in Table 3, the results of the total sample confirmed that: (1) The total effect of hope on life satisfaction was significant (*β* = 0.650, *p* < 0.001), and after the mediators entered the regression, the direct effect of hope on life satisfaction was still significant (*β* = 0.354, *p* < 0.001), (2) Hope positively predicted positive coping (*β* = 0.662, *p* < 0.001), as well as perceived social support (*β* = 0.145, *p* < 0.05), (3) Positive coping positively predicted perceived social support (*β* = 0.606, *p* < 0.001), (4) Positive coping positively predicted life satisfaction (*β* = 0.240, *p* < 0.01), and (5) Perceived social support positively predicted life satisfaction (*β* = 0.251, *p* < 0.001).

**Table 3:** Regression analysis results.

Regression		Model index		Coefficients	
Outcome variables	Independent variables	*R^2^*	*F*	*β*	*t*
Positive coping	Hope	0.44	170.76^***^	0.662	13.07^***^
Perceived social support	Hope	0.71	110.86^***^	0.145	2.27^*^
	Positive coping			0.606	9.53^***^
Life satisfaction	Hope	0.73	84.93^***^	0.354	5.69^***^
	Positive coping			0.240	3.29^**^
	Perceived social support			0.251	3.84^***^

* *p* < 0.05;

** *p* < 0.01;

*** *p* < 0.001.

*N* = 221. *β* = standardized coefficients.

Next, serial mediation analysis using the Bootstrap (model 6, sampling 5,000 times) method was conducted to examine the indirect effects of hope on life satisfaction. As shown in Table 4, the results indicated that the total indirect effect of hope on life satisfaction in the full model was significant −0.296, which was significant with a 95% CI [0.18, 0.41]. Specifically speaking, (1) the indirect effect of hope → positive coping → life satisfaction was significant (effect = 0.159, 95% CI [0.07, 0.26]). (2) The indirect effect of hope → social support → life satisfaction was not significant (effect = 0.036, 95% CI [−0.01, 0.09]), and (3) the indirect effect of hope → positive coping → social support → life satisfaction was significant (effect = 0.101, 95% CI [0.03, 0.17]).

**Table 4:** Total, direct and indirect effects of hope on life satisfaction.

Paths	Full	
	Effect	95% CI
Total effect	0.650	[0.84, 1.15]
Direct effects	0.354	[0.35, 0.73]
Indirect effect		
Total indirect effects	0.296	[0.18, 0.41]
Hope → Positive coping → Life satisfaction	0.159	[0.07, 0.26]
Hope → Social support → Life satisfaction	0.036	[−0.01, 0.09]
Hope → Positive coping → Perceived social support → Life satisfaction	0.101	[0.03, 0.17]

*N* = 221. Bootstrap sample size = 5,000. CI, confidence interval.

**Figure 2:**
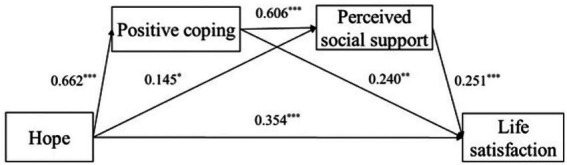
Associations between hope and life satisfaction for the whole sample (*N* = 221). Coefficients are standardized. ^*^p < 0.05, ^**^p < 0.01, ^***^p < 0.001.

Those findings indicated that hope could indirectly predict life satisfaction both through the mediating effect of positive coping and the chain intermediary of social support and positive coping. The final model for the whole sample was displayed in Figure 2.

## Conclusion and discussion

This study was to develop a model that elucidates the relationship between hope and life satisfaction, including the mediating role of the psychological predictors (i.e., positive coping and perceived social support) in the association between hope and life satisfaction of Chinese SEI tutors. The results suggested that hope, as a positive disposition, can improve life satisfaction of SEI tutors by promoting positive coping and perceived social support, where hope played a role through the chain mediating role of positive coping and perceived social support. Our findings were consistent among multiple measures of psychological health (Chen et al., 2021; Karataş et al., 2021). With Chinese SEI tutors faced with high unemployment rates and uncertain career development as participants, the current study extended the literature by demonstrating the role of positive coping and perceived social support in mediating the association between hope and life satisfaction, both, respectively, and through their sequential mediation effect. The findings revealed that positive coping and perceived social support were positively associated with hope and life satisfaction, which echoed the previous study about the relationship between hope and life satisfaction in college students and elderly individuals (Özdemir et al., 2022). This study confirmed the argument that people with hope perceive higher life satisfaction than those who are less hopeful (Nie et al., 2019), which supports Hypothesis 1.

Of particular interest in the study was the mediating role of positive coping and perceived social support between hope and life satisfaction. As noted earlier, we know of no other studies that examined this. The findings supported three hypothesized indirect effects: (1) hope → positive coping → life satisfaction, (2) hope → perceived social support → life satisfaction, and (3) hope → positive coping → perceived social support → life satisfaction. Furthermore, the study explained the mediated effect of positive coping and perceived social support between hope and life satisfaction. The findings extended the research on the mediation effects of positive coping and perceived social support in the positive psychology literature (Swanson et al., 2018; Wang et al., 2018).

By analyzing the impact of hope on life satisfaction through positive coping, we found that hope can improve the life satisfaction of SEI tutors by adopting positive coping, thus Hypothesis 2 was supported. Likewise, research confirmed that hope levels were positively associated with positive coping styles, including positive reappraisal, planning, acceptance, and fighting spirit. Positive coping mediates not only self-compassion and life satisfaction, but also self-efficacy and work engagement (Dunkley et al., 2016; Li et al., 2021; Mikus and Teoh, 2021). Those studies highlighted the importance of positive coping in promoting hope and life satisfaction. In general, this study reveals the mediating effect of positive coping as more hopeful individuals tend to adopt positive coping strategies, which helps to improve their life satisfaction. For SEI tutors with high hopes, positive coping styles can help them get through tough times, even when career pressures are overwhelming. Adopting positive coping strategies and channeling positive emotions significantly impacted work engagement, and active coping can improve life satisfaction (Li et al., 2013). This finding was consistent with several previous studies that positive coping not only helps achieve goals, obtain more resources, and relieves depression, but also improves individuals’ perception of life satisfaction (Schwarzer and Knoll, 2003).

Unique to this study was that it hinted at the sequential mediation effect of positive coping and perceived social support. Nie et al. (2019) stated that hope served as a partial mediator between social support and life satisfaction. This study further found that social support, with positive coping, mediates the association between hope and life satisfaction. Our findings supported Hypothesis 4 regarding the indirect effect of hope on life satisfaction through the chain mediating effect of positive coping and perceived social support, with the result being statistically significant. Hope is the enhancement of well-being in life through positive coping and perceived social support. For education workers, the more hopeful people are, the more active coping strategies they use, and the more likely they will enhance emotional bonds and gain more social support to complete the mediating mechanisms that enhance well-being (Xiang et al., 2020). The results of this study revealed that SEI tutors with higher hope are more likely to adopt positive coping strategies, such as being optimistic, taking the initiative to engage in problem-solving activities, or seeking help from others, which is more conducive to gaining strong social support, and, in turn, leads to life satisfaction. It echoed Swanson et al.’s (2018) finding that in addition to the direct effect of positive coping on life satisfaction, problem-oriented coping strategies positively affect social support that predicts satisfaction. In other words, more solid evidence in future studies will verify the debates about hope and life satisfaction of the SEI tutors.

## Practical implications

The current findings could help SEI tutors and people whose industry is undergoing significant changes to maintain mental health. Driven by the market demands, shadow education and private tutoring sprouted in many countries (Zhang and Bray, 2021), and thus the well-being of SEI tutors require equal attention, similar to their colleagues in public schools. With increasing research paying attention to the study of positive aspects of mental health in the post-COVID-19 era, it shows a similar call for positive tutoring that emphasizes applying the ethics of positive psychology to foster a positive learning environment in shadow education. Spiritual well-being was significantly associated with life satisfaction and hope (Özdemir et al., 2022). Therefore, this study revealed that despite the challenges and uncertainty about jobs and careers in the post-COVID-19 era, hope, as an alternative, affects the SEI tutors’ life satisfaction *via* positive coping and social support. Concerning the effect of hope on life satisfaction, one can argue that SEI tutors who took more positive coping strategies will have more opportunities to obtain social support and feel a stronger sense of happiness and contentment. Otherwise, they will confront difficulties in getting enough social support. Accordingly, this study sheds light on how SEI tutors could be supported to lead more satisfying life to combat the identity and industry crisis (Trent, 2016). The findings demonstrated the mediation effect of positive coping and perceived social support. Developing the psychological capital dimensions and positive coping through interventions, such as building social networks, caring, and various other supportive measures can be a valuable avenue to increase SEI tutors’ well-being. Moreover, SEI tutors are encouraged to actively engage in professional development to enhance their professional competitiveness radically.

This study can be insightful for the sustainable development of the shadow education industry by providing regulation implications for policymakers and SEI owners, especially during the implementation of the current shadow education regulatory policy. It is plausible that tutors who are hopeful about their career prospect and able to take advantage of positive coping will have a high level of life satisfaction, which, in turn, enhance their job satisfaction. As stated earlier, hope is regarded as both a trait and a state, people with hope perceive higher life satisfaction over time (Nie et al., 2019), and hope functions as the “engine” in the proposed model, transferring energy from positive coping and perceived social support to life satisfaction in SEI tutors. The results imply that hope is valued by tutors who act as teachers and work in very demanding environments as a proactive personality (Mikus and Teoh, 2021). Alternatively, we could interpret the results as suggesting that people with hope and positive coping are more likely to be drawn to the tutoring profession, and less likely to turn over. Emotional exhaustion and personal accomplishment are proven factors that influence life satisfaction through job satisfaction (Kanadli et al., 2022). As teachers’ teaching-specific hope acts as the predictor of personal responsibility (Eren, 2017), promoting tutors’ perception of hope increases their work engagement and performance and decreases the attrition rate. Given the role of perceived social support plays in the mechanism between hope and life satisfaction, we believed that government must pay attention to the social welfare of SEI tutors and provide alternative career paths for unemployed tutors to raise their hope and life satisfaction.

## Limitations and future directions

This study was limited in some perspectives. First, the sample size hinders the research, as the small number of Chinese SEI tutors recruited *via* online enrolment in Hangzhou could hardly represent all SEI tutors in China. Future research should contain more convincing samples, especially former SEI tutors who switched careers, to better support the present findings and might provide further insight into the study on the life satisfaction of tutors. Second, the cross-sectional approach fails to deduce a causal explanation. Therefore, longitudinal study in the future needs to elucidate the causal relationship between hope and life satisfaction. Third, this study has methodological limitations, namely, the process cannot produce a model that matches the data. Future scholars are recommended to increase their samples and employ structural equation model tools, especially those capable of testing the serial mediation model, including Amos and Lisrel. Fourth, the study does not consider other factors that may affect life satisfaction, such as career prospects, work relationships, and salary. Hence we recommend scholars examine other positive psychological variables in future research.

Despite the limitations, this study reveals the mechanism between hope and life satisfaction in SEI tutors *via* mediate testing. It further contributes to providing an alternative perspective to examine the industry of shadow education. The findings demonstrate that hope can promote SEI tutors to adopt positive coping styles and strengthen their perceived social support. The perceived social support can elevate their life satisfaction. In the future, researchers may investigate the variations of social support methods (for instance, occupational security and financial support) and numerous coping styles that affect life satisfaction among SEI tutors.

## Data availability statement

The raw data supporting the conclusions of this article will be made available by the authors, without undue reservation.

## Ethics statement

The studies involving human participants were reviewed and approved by the Institutional Ethics Committee of Jing Hengyi School of Education, Hangzhou Normal University. The patients/participants provided their electronic informed consent to participate in this study.

## Author contributions

MZ and YW: conceptualization, data curation, funding acquisition, and validation and methodology. MZ, JJ, YW, and LZ investigation, formal analysis, writing–original draft, writing review and editing. MZ: project administration. All authors contributed to the article and approved the submitted version.

## Funding

This research was funded by a general project of the National Social Science Fund of China, grant number BGA180053.

## Conflict of interest

The authors declare that the research was conducted in the absence of any commercial or financial relationships that could be construed as a potential conflict of interest.

## Publisher’s note

All claims expressed in this article are solely those of the authors and do not necessarily represent those of their affiliated organizations, or those of the publisher, the editors and the reviewers. Any product that may be evaluated in this article, or claim that may be made by its manufacturer, is not guaranteed or endorsed by the publisher.

## Footnotes

^1^ https://www.wjx.cn
